# Analysing the daily, weekly and yearly cycles of births and their implications for the NHS using linked data

**DOI:** 10.23889/ijpds.v1i1.298

**Published:** 2017-04-19

**Authors:** Gill Harper, Alison MacFarlane, Nirupa Dattani, Miranda Dodwell, Rod Gibson, Mario Cortina, Peter Martin

**Affiliations:** City University; Rod Gibson Associates Ltd; University College London

## Objectives

This project builds on previous work linking routinely collected data from birth registration, birth notification, death registration and hospital discharges, extending it to six million births in England and Wales from 2005 to 2014. This linkage is creating a new dataset to investigate previously unanswerable questions about variations in time of birth and its outcome which are highly contested in the health service in England and Wales:

1. How do numbers of births vary according to time of day, day of the week and time of year of birth and how does this relate to modes of onset of labour and delivery?
2. How do patterns of birth vary between maternity services in relation to variations in medical and midwifery staffing, patterns of intervention and size of unit?
3. How does the outcome of pregnancy, in terms of mortality and morbidity rates at birth and in the first year of life, vary according to time of birth in relation to gestational age, and intervention in the onset of labour and delivery?
4. Have the patterns changed over the years 2005 to 2014?

## Approach

Routinely collected national datasets for 2005-14 have been linked using patient identifiable data items. They differ in the way data items are recorded. Methods of linkage, quality assurance and data cleaning have been improved, compared with those developed in the original project.

This unique approach links clinical data from hospital admissions with more reliable data on birthweight from birth registration and with gestational age and time of birth from birth notification. This enables investigation of the associations between day and time of birth with its outcome.

This is also the first study to link mothers' and babies' hospital discharge data for England and Wales and to draw on public and patient involvement to include outcome measures specifically designed to be women-centred. Overall, the linkage has created a fuller range of data.

## Results

Results will be available by the time of the conference. Initial results already clearly illustrate differences in timings of birth by time of day and day of the week by mothers' age, gestational age and birth setting and for singleton and multiple births. 

## Conclusion

The study has created a valuable linked dataset that will uniquely enable analyses of associations between timing of birth and its outcome. These results will potentially be able to inform and impact NHS service provision.

